# From bad to worse: examining the deteriorating labour market outcomes of international graduates in Australia

**DOI:** 10.1007/s12546-022-09291-7

**Published:** 2022-08-23

**Authors:** Angelina Tang, Francisco Perales, Francisco Rowe, Janeen Baxter

**Affiliations:** 1 Institute for Social Science Research, The University of Queensland, Brisbane, Australia; 2 Australian Research Council Centre of Excellence for Children and Families over the Life Course, The University of Queensland, Brisbane, Australia; 3 School of Social Science, The University of Queensland, Brisbane, Australia; 4 Geographic Data Science Lab, Department of Geography and Planning, University of Liverpool, Liverpool, UK

**Keywords:** Australia, International graduates, Labour market, Skill utilisation, Skilled migration, Study-to-work transition

## Abstract

International graduates have been shown to experience comparatively poor outcomes in their transition to the host labour market after course completion. In Australia, international graduates typically fare worse than domestic graduates in the labour market, with new evidence pointing to a deterioration in their relative labour market position over the years. The contributing factors for the deterioration, however, remain unclear. In this article, we analysed unique large-scale survey data from the Australian Graduate Survey to identify the factors underlying the deteriorating labour market outcomes of international graduates from 2000. Our findings indicate that the deteriorating labour market outcomes of international graduates can be largely linked to the declining share of these graduates who are a citizen or permanent resident of Australia. The rising percentage of international graduates who are non-native English-speakers also played a role, albeit a modest one. These findings call attention to the persistent labour market disadvantage of international graduates and highlight the inadequacies of existing policies aimed at temporary retention and stronger English language skills in promoting labour market integration.

## Introduction

Over the past two decades, both industrialised and industrialising countries have reported strong growth in their demand for high-skilled migrants in an effort to attain and maintain a competitive edge in the global economy against a backdrop of fertility decline and population ageing (Hugo, 2006; Suter and Jandl 2008; Shachar & Hirschl, 2013). As part of this ‘global quest for talent’ (Kuptsch & Pang, 2006, p. 1), several countries have turned to international students graduating from their local higher education institutions (hereinafter referred to as *international graduates*), who are generally deemed a convenient source of young and employable high-skilled migrants (Hawthorne, 2005; Hugo, 2006; Suter and Jandl 2008). Australia, in particular, is recognised as a front-runner in the retention of international graduates for introducing and maintaining post-study migration and employment pathways since 1999 (Peykov, 2004; Gribble & Blackmore, 2012). Amongst other things, it was believed that international graduates would make ‘immediate positive contribution to the Australian economy, labour market and budget’ (DIMA 1999, p. vii, cited in Hawthorne 2010).

Nonetheless, incipient evidence paints a grim picture of the labour market outcomes of international graduates who remained in Australia after the completion of their studies (Birrell et al., 2006; Hawthorne, 2010; Trevelyan & Tilli, 2010; Li & Miller, 2013; Hawthorne & To, 2014; Faggian et al., 2016). Specifically, international graduates tend to perform poorly in the Australian labour market compared to local graduates and high-skilled migrants recruited offshore. Economic inactivity, unemployment, part-time employment and education-job mismatch are prevalent amongst international graduates, as well as low job satisfaction and wage returns. Importantly, recent work reveals that their labour market outcomes have deteriorated since the introduction of post-study migration and employment pathways in 1999 (Tang et al., 2022). As a result, the gaps in the labour market outcomes of international and domestic graduates have widened over the years (Tang et al., 2022).

The accumulating evidence has prompted a growing interest in the factors shaping the comparatively poor outcomes of international graduates in the Australian labour market. In particular, the below-par labour market outcomes have been mainly attributed to a general lack of English language proficiency, permanent residency status, work readiness and local discipline-related work experience amongst international graduates (Birrell and Healy 2008; Birrell et al., 2009; Li & Miller, 2013; Hawthorne & To, 2014; Blackmore et al., 2017). However, the existing understanding is largely drawn from qualitative research on selected subpopulations and specific years. Consequently, results are not generalisable to the wider international graduate population. Furthermore, the factors contributing to overall worsening labour market position for international graduates are not yet clear.

To help address these research gaps, we empirically examine the forces influencing the labour market outcomes of international graduates who remained in Australia after the introduction of post-study and migration pathways in 1999. Drawing on the Australian Graduate Survey (AGS), we use the Blinder-Oaxaca decomposition technique to explain the deteriorating labour market outcomes of international graduates between 2000 and 2015^1^. We also seek to identify the main factors contributing to the gaps in the labour market outcomes of international and domestic graduates in 2015.

The remainder of the article is divided into five sections. We first provide a review of existing literature on the retention of international graduates in Australia, with an emphasis on factors thought to influence their transition to the host labour market after course completion. We then describe the data and methods employed to examine their unfavourable labour market position, followed by a discussion of the results. In the last section, we discuss the significance of the present study and its policy implications.

## Barriers to the labour market integration of international graduates in Australia

In the early years of opening up its education services to the international market, Australia had no clear post-study migration and employment pathways for its international graduates. In view of rising global competition for high-skilled migrants, Australia introduced several changes to its immigration policies at the turn of the 21st century to retain international graduates following the completion of their studies. Specifically, exemptions were put in place to waive international graduates from the strict requirements of permanent residency application on discipline-related work experience and English language testing, along with bonus points for their Australian education credentials (Birrell and Healy 2008; Hawthorne, 2010). Dedicated immigration programmes mainly targeted at the temporary retention of international graduates were introduced over the 2000s, including the Temporary Graduate visa - subclass 485 (which was known as the Skilled Graduate visa prior to 2011).

These policy changes have drawn attention to the ability of international graduates to integrate into the Australian labour market. In recent years, a growing body of literature has attempted to explain the poor labour market outcomes of international graduates who remained in Australia following the completion of their studies. Focusing on the individual level, incipient evidence has largely pointed to inadequacies in their socio-demographic and skill characteristics. Specifically, it has been suggested that international graduates generally fall short of the requisites for a typical graduate recruit in Australia, which include advanced English language proficiency, permanent and unrestricted work rights, work readiness, and local discipline-related work experience.

### The Big Four in the employment of international graduates

Much existing work has attributed the poor labour market outcomes of international graduates to their low level of English language skills, particularly amongst the non-native English-speakers (Birrell, 2006; Birrell & Edwards, 2007; Birrell and Healy 2008; Hawthorne 2014). Recent studies suggest that non-native English-speaking international graduates are less likely to secure full-time employment and are more susceptible to qualification mismatch, compared to those with English as their main language (Li & Miller, 2013; Hawthorne & To, 2014). There is also some emerging evidence suggesting that English language competence - or a lack thereof - is the most important factor shaping the labour market outcomes of international graduates (Arkoudis et al., 2009; Hawthorne, 2010; Hawthorne & To, 2014). In addition to challenges with communication, poor command of English language may limit the accumulation of social capital that enhances the labour market prospects of international graduates, for example, building professional and social networks and locating suitable jobs that involve speaking English (Blackmore et al., 2017).

The literature has also identified the temporary residency status of many international graduates as an important barrier to their integration into the Australian labour market (Robertson 2014). For example, international graduates who hold a temporary visa tend to take up part-time jobs that do not align with their educational attainment, compared to those graduates who are a citizen or permanent resident of Australia (Li & Miller, 2013; Hawthorne & To, 2014). While temporary residents typically have full legal work rights, Australian employers customarily favour the employment of citizens and permanent residents in professional positions, including apprenticeships, internships and graduate programmes that traditionally form the key entry point into the Australian labour market (Hugo, 2006; Blackmore et al., 2014; Gregory, 2014). The hesitance to hire temporary residents is said to have channelled international graduates into occupational niches characterised by casual, low-skill, low-paying jobs and labour exploitation (Nyland et al., 2009; Robertson 2014). International graduates have also been found to prioritise their permanent residency applications at the expense of career progression mainly to attain a level playing field in the Australian labour market (Blackmore et al., 2014, 2017).

Furthermore, the literature has called attention to a general lack of work readiness amongst international graduates, a characteristic that has been broadly considered to be instrumental in a seamless transition into the Australian labour market. In particular, low levels of ‘soft’ skills - such as critical thinking and interpersonal skills - have been highlighted as a matter of concern, alongside the fit with the Australian work culture (Birrell & Edwards, 2007; Birrell and Healy 2008; Birrell et al., 2009; Blackmore et al., 2014). In light of higher education expansion and credential inflation, more employers are demanding well-rounded, work-ready graduates who can ‘hit the ground running’, with soft skills and cultural fit taken to indicate employability (Blackmore et al., 2014, p. 21). While educational credentials and sound academic achievements are now ‘taken as a given’, soft skills and cultural fit play a growingly important part in recruitment, especially at large and prestigious firms that attract significant numbers of graduates (Blackmore et al., 2014, p. 22).

International graduates also tend to lack local work experience, particularly experience relevant to their field of study (Birrell et al., 2009; Robertson et al., 2011). Many international graduates do not meet the requisite citizenship and residency status for apprenticeships, internships and graduate programmes critical to acquiring local, discipline-related work experience in Australia (Jackling, 2007; Blackmore et al., 2014). As pointed out earlier, international graduates mostly take up low-skill jobs that do not align with their educational attainment (Nyland et al., 2009; Robertson 2014). While some employers value this experience with the broader, local work environment, the others show concerns about inadequate knowledge of discipline-specific organisational practices (Blackmore et al., 2014, 2017). Further, many employers routinely recruit those who participated in their apprenticeships, internships and graduate programmes (Jackling, 2007). In contrast, the casual, low-skill jobs frequently occupied by international graduates present limited opportunities for progression to long-term, high-skill positions (Nyland et al., 2009; Robertson 2014; The Smith Family 2014).

Nonetheless, there has been growing concern about employers’ expectations of international graduates. Blackmore and colleagues (2014) argue that the requisites for citizenship and residency status, English language proficiency and subjective criteria such as cultural fit may be used deliberately to preclude international graduates from applying for jobs. These authors specifically draw attention to ‘an inherent degree of racism amongst middle management in Australia’ and ‘a high level of xenophobia in [the] Australian society’ (p. 23). Such prejudicial treatment has been linked to stereotypes of international graduates as ‘deficient workers’ (Robertson, 2011, p. 2206). Birrell and Healy (2008) indicate that ‘there is no doubt that employers currently hold negative views about the capabilities of former [international] students’ (p. 13). In particular, international graduates are not only seen as lacking English language competence and important soft skills, but also as being ill-fitted to the Australian work environment (Blackmore et al., 2014, 2017; Robertson 2014). The ‘migration hunter’ stereotype has further triggered ‘moral panic’ over ‘problematic and opportunistic’ international graduates who are ultimately interested in Australian citizenship or permanent residency (Robertson, 2011, p. 2197; Tran & Vu 2016, p. 204).

### Gaps in knowledge and evidence needs

In this respect, numerous policy changes have been put in place over the years to improve the labour market outcomes of international graduates. For example, the immigration framework shifted to favour employer sponsorship over independent applicants in 2009, along with stricter requirements on English language proficiency and discipline-relevant work experience (Hawthorne, 2010; Spinks, 2010, 2016; Phillips & Spinks, 2012). Further, the programmes targeted at temporary retention are designed to offer international graduates the opportunities to enhance their English language skills or discipline-relevant work experience before applying for permanent residency (Spinks, 2016).

As pointed out earlier, however, prior research has been largely limited to selected subpopulations, such as accounting, engineering or health graduates (Birrell and Healy 2008; Hawthorne, 2010; Hawthorne & To, 2014; Blackmore et al., 2014, 2017) and Chinese or Indian nationals (Hawthorne 2014; Blackmore et al., 2017). Much of the published work has also drawn on qualitative research such as interviews with international graduates or their current and prospective employers (Birrell, 2006, Birrell and Healy 2008; Birrell et al., 2009; Blackmore et al., 2014, 2017; Robertson 2014; Robertson et al., 2011; Tran & Vu, 2016). While these qualitative studies have provided invaluable insights into the labour market outcomes of international graduates in Australia, they are typically exploratory and descriptive in nature. Furthermore, the available studies have mostly adopted a static, cross-sectional perspective focusing on the outcomes of international graduates from a particular cohort (Hawthorne, 2010; Faggian et al., 2016) or the aggregate outcomes of several graduating cohorts over a relatively short period of time (Li & Miller, 2013; Hawthorne 2014; Hawthorne & To, 2014).

Taken together, the existing research has offered suggestive evidence and fragmented understanding of the labour market outcomes of international graduates who remained in Australia since the introduction of post-study migration and employment pathways in 1999. To address these gaps, we assess the labour market outcomes of international graduates who remained in Australia between 2000 and 2015. Specifically, we seek to identify the main factors contributing to the worsening labour market outcomes amongst international graduates and the gaps with domestic graduates. The following section describes the data and methods employed in this study.

## Data and methods

### The Australian Graduate Survey

To examine the labour market outcomes of international graduates in Australia, we utilise the AGS, an annual survey funded by the national education department to assess the career progression of tertiary students graduating from universities and a sample of other higher education institutions, primarily the Technical and Further Education institutions and colleges (GCA 2016). The AGS is the only nationally representative survey that systematically measures the study-to-work transition of tertiary graduates in Australia with a focus on their early labour market outcomes at approximately four months after course completion (GCA 2016). The survey records a response rate of 41–43% and 58–63% for international and local graduates, respectively, which is generally considered satisfactory for surveys administered to an entire population utilising paper and online forms. The target population for this study - that is, international graduates who remained in Australia following course completion - is most likely over-represented in the responses compared to international graduates who had left by the survey reference date and thus are more difficult to reach (GCA 2008). In this study, we specifically used the three waves of the survey conducted in 2000, 2008 and 2015, as will be discussed in more detail when introducing the methods.

### Identifying international and domestic graduates

We use survey information on fee-paying status to identify international and domestic graduates (GCA 2008). Specifically, international graduates include those who were wholly or mainly an international fee-paying student for the tertiary education they had just completed, while domestic graduates are those who were wholly or mainly an Australian fee-paying student or a recipient of government assistance under the Higher Education Contribution Scheme loan programme. Following the convention in labour market studies (OECD 2014), two groups of individuals are excluded from the analyses: (1) those who are typically not available for or not fully committed to work, specifically, those who fall outside the working age of 15 to 64 years or are pursuing further study; and, (2) those who worked in occupations with poorly defined skill requirements, specifically, legislators, members of the armed forces, and self-employed individuals.

### Measuring labour market outcomes

We examine four labour market indicators: economic inactivity, unemployment, part-time employment, and education-job mismatch. Respondents who were not engaged in nor actively seeking paid work are categorised as economically inactive. Economically active respondents are divided into those who were not engaged in but were actively seeking paid work (i.e., unemployed), and those who were participating in paid work (i.e., employed). The latter are further broken into part- and full-time employed. We follow the Organisation for Economic Co-operation and Development (OECD) (2014) and examine three types of mismatch: (1) qualification mismatch - to capture misalignment to level of education; (2) field-of-study mismatch - to capture misalignment to field of study; and (3) skill mismatch - to capture misalignment to non-technical skills.

To identify qualification mismatch, we employ the job analysis method using the Australian and New Zealand Standard Classification of Occupations (ANZSCO), First Edition, Revision 1 (ABS 2009) as the analytic framework. Specifically, the highest level of education for the respondents is compared to that expected for their main occupation at the broadest level of the ANZSCO (i.e., Major Groups, 1-digit level). Respondents are considered mismatched if their educational attainment did not match that prescribed for their occupation. In this study, we focus on over-qualified graduates (i.e., those who held a higher level of education than the prescribed level) given the greater prevalence and negative effects of this mismatch (Hartog, 2000; Fleming & Kler, 2008; Li 2013).

We further employ the job analysis method to capture field-of-study mismatch. Specifically, we follow the OECD (2014) and utilise an analytic framework developed by Wolbers (2003) to determine the alignment between field of study and occupation drawing on the International Standard Classification of Occupations 1998 and the International Standard Classification of Education 1997. In this study, the analytic framework was adopted to identify appropriate occupations at the Unit Group (4-digit) level of the ANZSCO for each field of study at the Broad Field (2-digit) level of the Australian Standard Classification of Education. Respondents who did not work in an occupation deemed suitable for their field of study are considered to be mismatched.

For skill mismatch, we use the direct self-assessment method and analyse graduate perceptions of the importance of non-technical skills acquired during the recent course to their employment. In this case, respondents are considered mismatched if they indicated that these skills were not important to their employment. In contrast, those who responded formal requirements were important or somewhat important, are categorised as matched.

### Blinder-Oaxaca decomposition

The Blinder-Oaxaca decomposition is a technique that is widely employed to examine differences in an outcome between two groups; originally, ethnic and gender disparities in wages (Blinder, 1973; Oaxaca, 1973). The fundamental principle of this decomposition technique is that the difference can be broken down into two components: a characteristics effect and a coefficients effect. The *characteristics effect* captures differences in the composition of the two groups. For instance, higher wages received by white men, compared to black men, in the United States could be attributed to their higher level of educational attainment (Blinder, 1973). On the other hand, the *coefficients effect* measures differences in the effects of individual characteristics. Using the same example, wage advantages amongst white men could be linked to a higher rate of return on their education (Blinder, 1973). The coefficients effect is usually interpreted as evidence of differential treatment or discrimination; however, it may also reflect other behavioural responses, for instance, individual aspirations.

The technique begins by modelling the dependent variable ($Y$) as a function of a set of predictors ($X$) and their associated regression coefficients ($\beta$) for the two groups ($i$) respectively:

1 $${Y}_{i}=F\left({X}_{i}{\beta }_{i}\right)$$

The estimated mean difference between the groups - say, $A$ and $B$ - is then decomposed into an overall characteristics effect and an overall coefficients effect, as follows:

$${\overline{Y}}_{A}- {\overline{Y}}_{B}= \overline{F\left({X}_{A}{\beta }_{A}\right)} - \overline{F\left({X}_{B}{\beta }_{B}\right)}$$

2 $${\overline{Y}}_{A}- {\overline{Y}}_{B}= \left[\overline{F\left({X}_{A}{\beta }_{A}\right)} - \overline{F\left({X}_{B}{\beta }_{A}\right)}\right] + \left[\overline{F\left({X}_{B}{\beta }_{A}\right)} - \overline{F\left({X}_{B}{\beta }_{B}\right)}\right]$$

The first square bracket on the right-hand side of Eq. (2) captures the overall characteristics effect. It estimates how disparities in the distribution of characteristics between the groups (i.e., ${\overline{X}}_{A}$ and ${\overline{X}}_{B})$ influence the mean group difference while holding the associated regression coefficients constant (i.e., ${\beta }_{A}$). The second square bracket measures the aggregate impact of varying effects (i.e., ${\overline{\beta }}_{A}$ and ${\overline{\beta }}_{B})$ on the same characteristics (i.e., ${X}_{B}$), or the overall coefficients effect. Group $A$, the comparison group, typically represents the group with a higher average outcome, whereas Group $B$, the reference group, captures the group with a lower average outcome.

In this study, we perform two decomposition analyses. The first analysis focuses on the *deterioration* in the labour market outcomes of international graduates, while the second analysis examines the *gaps* in the labour market outcomes of international and domestic graduates in the most recent year of data. Both analyses take into consideration four labour market indicators - specifically, economic inactivity, unemployment, part-time employment and education-job mismatch - which are modelled as binary dependent variables. In this respect, we apply a version of the detailed decomposition technique adapted to a logistic regression framework based on Yun (2004, 2008).^2^

Our analyses consider ten variables: age, gender, language background, disability status, country of permanent residence, highest level of education, field of study, overall course satisfaction, paid work experience during the final year of study, and employment location (see Appendix - Table A for a detailed description of the variables). Given emerging evidence and recent debates, particular attention is placed on the variables capturing the influence of language background, country of permanent residence and paid work experience during the final year of study. The variable country of permanent residence is omitted from the second analysis as domestic graduates are not required to identify their country of permanent residence in the AGS.

Table 1 summarises the comparison groups and reference groups for both decomposition analyses. It is important to note that the models studying temporal changes in economic inactivity, unemployment, part-time employment and qualification mismatch focus on the shifts between 2000 and 2015, whereas the models on field-of-study mismatch and skill mismatch examine a shorter period between 2008 and 2015. Consistent educational and occupational data required to measure field-of-study mismatch are only available from 2006 due to changing coding schemes over the period of analysis, and the survey question capturing skill mismatch was introduced in 2008. For comparability and ease of interpretation, the models analysing temporal changes in field-of-study mismatch and skill mismatch cover the period after 2008.

**Table 1:** Comparison and reference groups

Analysis	Dependent variable	Comparison	Reference
#1 Deterioration in the labour market outcomes of international graduates	Economic inactivity Unemployment Part-time employment Qualification mismatch	International graduates in **2015** (Base n = 8,319)	International graduates in **2000** (Base n = 3,358)
	Field-of-study mismatch Skill mismatch	International graduates in **2015** (Base n = 8,319)	International graduates in **2008** (Base n = 4,459)
#2 Gaps in the labour market outcomes between international and domestic graduates	Economic inactivity Unemployment Part-time employment Qualification mismatch Field-of-study mismatch Skill mismatch	International graduates in **2015** (Base n = 8,319)	Domestic graduates in **2015** (Base n = 62,342)

## Factors driving the deterioration in the labour market outcomes of international graduates

Table 2 presents summary statistics for the first decomposition analysis that addresses the deteriorating labour market outcomes of international graduates. The results show a clear trend of deteriorating labour market outcomes amongst international graduates who remained in Australia between 2000 and 2015. Part-time employment and qualification mismatch grew by at least 40% points from approximately 15% in 2000 to more than 56% in 2015. Meanwhile, economic inactivity saw a sizeable increase from 3.6 to 14.8%, whereas unemployment went from 9.8 to 32.7%. On the other hand, field-of-study mismatch rose slightly from 28.2% to 2008 to 33.5% in 2015, as did skill mismatch - from 16.2 to 19.1%.

The results shown in Table 2 also reveal important changes to the socio-demographic and skill profile of international graduates who remained in Australia between 2000 and 2015. Specifically, the results show a substantial drop in the percentage of international graduates who were a citizen or permanent resident of Australia (75.3% points), as well as those who took up paid work during the final year of study (35.6% points). Importantly, these declines remained substantial between 2008 and 2015 (9.6 and 9.9% points, respectively). On the other hand, there were sizable increases between 2000 and 2015 in the share of non-native English-speakers (61.0% points), Chinese nationals (30.0% points) and postgraduate degree holders (29.4% points). Such growth was also documented between 2008 and 2015, although the rise in the share of non-native English-speakers was relatively small (4.2% point).

**Table 2:** Summary statistics

Variable	Percentage (%)			Difference (pp)	
	**(a)**	**(b)**	**(c)**	**(c) - (a)**	**(c) - (b)**
	2000	2008	2015	2015 − 2000	2015 − 2008
**Dependent variables**					
**Labour market indicators**					
Economic inactivity	3.6		14.8	11.2	
Unemployment	9.8		32.7	22.9	
Part-time employment	14.1		58.3	44.2	
Qualification mismatch	15.1		56.0	40.9	
Field-of-study mismatch		28.2	33.5		5.3
Skill mismatch		16.2	19.1		2.9
**Independent variables**					
Age					
*Less than 25*	49.0	41.6	34.5	-14.5	-7.1
*Aged 25–34*	32.8	54.0	60.2	27.4	6.2
*35 and above*	18.2	4.4	5.3	-12.9	0.9
Male	39.5	50.4	47.3	7.8	-3.1
Non-English-speaking background	20.5	77.3	81.5	61.0	4.2
Disability	4.2	0.8	1.3	-2.9	0.5
Country of permanent residence					
*Australia*	86.8	21.1	11.5	-75.3	-9.6
*China*	1.1	21.3	31.1	30.0	9.8
*Hong Kong*	1.0	4.1	3.2	2.2	-0.9
*India*	1.1	13.6	10.1	9.0	-3.5
*Indonesia*	1.4	4.0	3.0	1.6	-1.0
*Malaysia*	1.8	7.6	6.2	4.4	-1.4
*Singapore*	2.4	3.0	2.2	-0.2	-0.8
*Europe*	0.6	4.3	3.1	2.5	-1.2
*South-East Asia*	0.7	3.8	7.8	7.1	4.0
*North-East Asia*	1.6	4.5	3.5	1.9	-1.0
*Southern and Central Asia*	0.3	5.1	8.3	8.0	3.2
*Northern, Central and South America*	0.2	3.3	4.0	3.8	0.7
*Africa, Middle East and Oceania*	1.0	4.4	5.9	4.9	1.5
Graduate degree	30.3	52.6	59.7	29.4	7.1
Field of study					
*Sciences and Environmental Studies*	5.8	4.3	3.9	-1.9	-0.4
*Information Technology*	8.0	14.0	12.1	4.1	-1.9
*Engineering and Related Technologies*	6.7	8.4	11.2	4.5	2.8
*Architecture and Building*	5.1	2.0	2.6	-2.5	0.6
*Health*	13.5	9.6	12.7	-0.8	3.1
*Education*	16.0	2.7	3.1	-12.9	0.4
*Management and Commerce*	25.9	49.3	46.3	20.4	-3.0
*Society and Culture*	10.3	5.8	5.4	-4.9	-0.4
*Creative Arts and Services*	8.6	3.9	2.6	-6.0	-1.3
Unhappy	10.2	13.9	6.9	-3.3	-7.0
Paid work experience	82.9	57.2	47.3	-35.6	-9.9
Metropolitan locations	87.4	94.0	93.5	6.1	-0.5

**Notes**: *pp* stands for percentage point. The numbers may not add up due to rounding.

**Source**: Australian Graduate Survey, 2000, 2008 and 2015

**Fig. 1:**
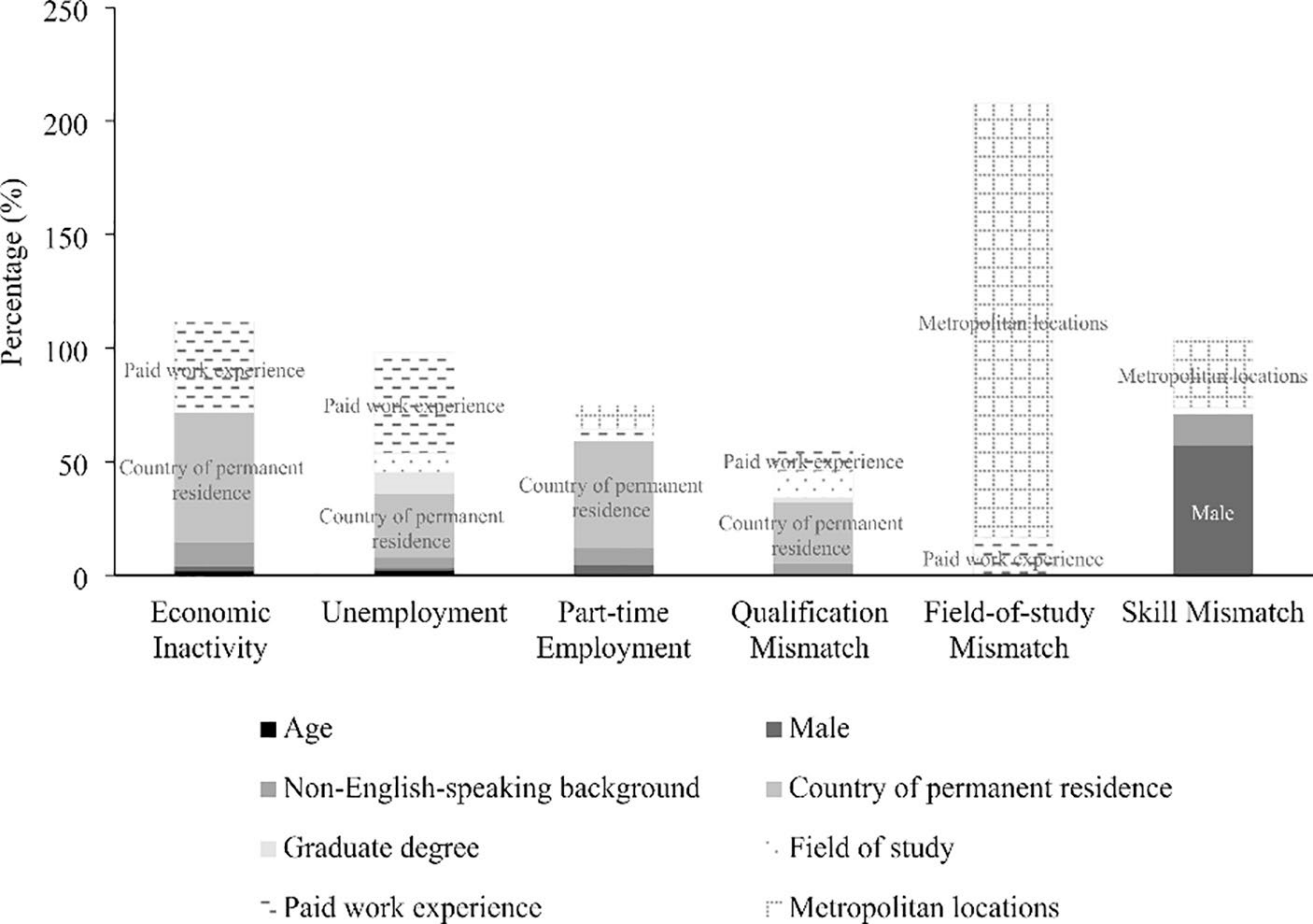
Main factors driving the deterioration in the labour market outcomes of international graduates **Notes** The figure shows the net positive contribution of the main drivers of changes in the respective labour market indicator combining both the characteristics effect and the coefficients effect. The analyses on field-of-study mismatch and skill mismatch are restricted to a shorter timeframe due to limitations in data availability.

Figure 1 summarises the main factors driving the deterioration in the labour market outcomes of international graduates between 2000 and 2015. Of the ten variables included in the models, country of permanent residence and paid work experience appear to be the key contributing factors for the deteriorating labour market outcomes amongst international graduates over the observation period. These variables, however, seem less relevant to field-of-study mismatch and skill mismatch, possibly due to the different period of analysis covered for these outcomes (i.e., between 2008 and 2015). Instead, the rising rates of field-of-study mismatch and, to a smaller extent, skill mismatch can be attributed to employment location.

The full detailed decomposition estimation presented in Table 3 sheds further light on the mechanisms through which these factors shaped changes in the labour market outcomes of international graduates between 2000 and 2015. The top row shows the aggregated contribution of the characteristics effects and coefficients effects to the labour market indicators for the respective column. The rows below provide the individual contribution of each predictor to these effects. A positive value denotes an effect that contributes to the deterioration in the respective labour market indicator, while a negative value shows a counterbalancing effect that offsets the deterioration. At an aggregate level, the deterioration in economic inactivity and unemployment can be mainly attributed to compositional changes amongst international graduates over the period of analysis. In contrast, rising part-time employment and education-job mismatch were largely linked to coefficients effects - or changing behavioural responses of, or towards, the international graduate population over the observation period.

**Table 3:** Detailed decomposition estimation of the deterioration in the labour market outcomes of international graduates between 2000 and 2015

Independent variables	Dependent variables											
	**Characteristics effect**						**Coefficients effect**					
	2000–2015				2008–2015		2000–2015				2008–2015	
	INAC	UNEMP	PTEMP	QMM	FOSMM	SMM	INAC	UNEMP	PTEMP	QMM	FOSMM	SMM
**Aggregate effect**	-91.2	-73.2	29.9	34.0	2.9	-62.5	-08.8	-26.8	70.2	66.0	97.1	162.5
**Individual variables**												
Age	-00.8	0-0.1	-0.2	0.1	-0.2	-4.3	-01.0	-02.0	-0.1	-3.2	-13.0	-14.2
*Less than 25*	*0.9*	*-1.4*	*0.1*	*-0.7*	*-0.4*	*-1.7*	*1.6*	*1.6*	*-2.3*	*-6.6*	*5.5*	*26.1*
*Aged 25–34*	*0.5*	*0.1*	*-0.1*	*0.1*	*0.4*	*-3.3*	*0.0*	*2.1*	*3.1*	*1.8*	*-19.5*	*-40.9*
*35 and above*	*-0.6*	*1.2*	*-0.1*	*0.7*	*-0.2*	*0.7*	*-0.6*	*-1.8*	*-0.8*	*1.6*	*1.0*	*0.6*
Male	0-0.2	-00.6	-0.3	-0.3	-4.0	-3.0	-02.1	-00.5	4.8	-2.0	-31.1	60.2
Non-English-speaking background	-11.8	-05.3	10.1	7.4	-1.5	2.9	0-0.7	0-0.4	-2.8	-2.1	-38.1	11.0
Disability	0-0.3	0-0.1	0.2	0.6	-0.7	-0.7	0-0.2	-00.3	-1.5	-1.8	-3.2	-3.6
Country of permanent residence	-44.3	-02.8	29.7	23.4	21.3	-10.0	-12.4	-25.0	17.5	3.5	-29.6	1.5
*Australia*	*39.2*	*0.3*	*23.5*	*20.0*	*30.8*	*14.3*	*12.1*	*25.4*	*17.8*	*3.0*	*-32.1*	*16.8*
*China*	*7.6*	*4.9*	*2.2*	*-2.7*	*-10.3*	*-19.4*	*0.0*	*0.4*	*-0.1*	*-0.3*	*-14.0*	*-45.9*
*Hong Kong*	*0.5*	*0.2*	*0.5*	*0.3*	*-1.2*	*-0.8*	*-0.1*	*-0.2*	*0.1*	*0.0*	*1.1*	*-10.6*
*India*	*-3.3*	*-2.0*	*2.4*	*2.9*	*-2.9*	*-1.2*	*-0.1*	*-0.3*	*0.0*	*0.6*	*15.1*	*24.5*
*Indonesia*	*0.0*	*0.4*	*0.3*	*-0.1*	*-1.0*	*-2.3*	*0.0*	*-0.3*	*0.2*	*-0.1*	*1.3*	*0.5*
*Malaysia*	*0.7*	*0.2*	*-0.5*	*-0.2*	*1.4*	*-1.8*	*0.3*	*-0.3*	*-0.2*	*-0.2*	*-3.7*	*5.8*
*Singapore*	*0.0*	*0.0*	*0.0*	*-0.1*	*-0.1*	*0.5*	*0.2*	*-0.1*	*-0.2*	*0.6*	*1.2*	*0.5*
*Europe*	*0.2*	*-0.7*	*-1.0*	*-0.3*	*0.6*	*-1.0*	*0.0*	*0.1*	*0.0*	*0.1*	*2.5*	*5.3*
*South-East Asia*	*0.2*	*0.5*	*1.5*	*1.4*	*3.3*	*-2.1*	*0.0*	*0.3*	*-0.1*	*0.0*	*-0.7*	*-3.1*
*North-East Asia*	*0.2*	*0.3*	*-0.1*	*-0.2*	*-0.3*	*0.2*	*0.1*	*0.2*	*0.1*	*-0.5*	*1.0*	*-8.1*
*Southern and Central Asia*	*-1.4*	*-1.0*	*1.9*	*2.1*	*1.1*	*3.4*	*0.0*	*-0.1*	*0.0*	*-0.1*	*-4.7*	*3.7*
*Northern, Central and South America*	*-0.6*	*-0.9*	*-1.9*	*-0.8*	*-0.7*	*1.2*	*-0.1*	*0.0*	*-0.1*	*-0.1*	*0.9*	*8.2*
*Africa, Middle East and Oceania*	*1.0*	*0.5*	*0.9*	*1.0*	*0.6*	*-1.1*	*0.0*	*-0.1*	*0.0*	*0.5*	*2.3*	*3.7*
Graduate degree	0-6.1	-03.6	-2.7	-2.9	0.6	5.0	0-0.2	-06.1	-3.3	4.9	-31.3	-9.5
Field of study	0-3.7	-08.9	-1.7	9.1	-13.6	-25.9	-00.0	0-0.6	1.7	2.7	-47.3	-37.8
*Sciences and Environmental Studies*	*0.4*	*-0.3*	*-0.2*	*-0.3*	*-2.1*	*-2.2*	*-0.3*	*-0.6*	*-0.5*	*-0.6*	*7.6*	*13.7*
*Information Technology*	*-0.7*	*-0.3*	*-0.7*	*-0.3*	*-3.3*	*0.9*	*0.0*	*-0.1*	*0.3*	*1.2*	*-20.4*	*-43.2*
*Engineering and Related Technologies*	*-0.6*	*1.8*	*0.3*	*0.3*	*5.6*	*2.4*	*-0.2*	*1.8*	*3.0*	*1.1*	*25.8*	*16.4*
*Architecture and Building*	*-0.3*	*0.3*	*0.3*	*-0.1*	*0.8*	*-0.2*	*0.0*	*-0.9*	*-0.5*	*0.0*	*4.5*	*8.9*
*Health*	*0.1*	*0.2*	*-0.4*	*-1.4*	*-27.4*	*-14.6*	*1.2*	*1.9*	*-0.5*	*-0.2*	*-9.2*	*12.0*
*Education*	*-1.5*	*7.4*	*-1.8*	*4.8*	*-1.0*	*-5.5*	*1.3*	*1.4*	*1.0*	*1.7*	*0.4*	*-7.0*
*Management and Commerce*	*0.8*	*1.9*	*0.9*	*6.4*	*9.1*	*-3.7*	*-2.0*	*-3.3*	*2.3*	*3.2*	*-37.7*	*-21.5*
*Society and Culture*	*-1.4*	*-0.5*	*0.4*	*-0.2*	*0.2*	*-0.4*	*0.5*	*-1.2*	*-2.0*	*-3.6*	*-9.2*	*-12.8*
*Creative Arts and Services*	*-0.4*	*-1.7*	*-0.4*	*0.0*	*4.4*	*-2.6*	*-0.6*	*0.4*	*-1.5*	*-0.1*	*-9.0*	*-4.3*
Unhappy	-00.2	0-0.5	-0.1	-0.1	5.8	-23.1	0-0.5	-00.6	-0.5	0.3	-10.5	7.0
Paid work experience	-44.5	-52.7	-6.9	-4.4	-4.1	-2.0	0-4.5	0-8.3	12.3	14.9	20.9	4.6
Metropolitan locations			1.6	1.1	-0.7	-1.3			8.7	-33.2	191.7	32.1
Constants							0-0.7	-01.6	33.3	82.0	88.6	111.2

**Notes**: The abbreviations (INAC, UNEMP, PTEMP, QMM, FOSMM and SMM) denote, from left to right, economic inactivity, unemployment, part-time employment, qualification mismatch, field-of-study mismatch and skill mismatch. The numbers may not add up due to rounding. The logistic regression coefficients underlying the decomposition analysis are presented in Appendix - Table B. The analyses on field-of-study mismatch and skill mismatch are restricted to a shorter timeframe due to limitations in data availability.

**Source**: Australian Graduate Survey, 2000, 2008 and 2015

More specifically, the results show that the deterioration in the labour market outcomes of international graduates is in part linked to the influxes of non-native English-speakers, who have been previously found to perform poorly in the Australian labour market (Li & Miller, 2013; Hawthorne & To, 2014). Specifically, the 61% point rise in the share of non-native English-speakers between 2000 and 2015 explains an estimated 11.8% of the increase in economic inactivity, 5.3% for unemployment, 10.1% for part-time employment, and 7.4% for qualification mismatch. A small part of the rise in skill mismatch between 2008 and 2015 (2.9%) was attributed to the influxes of non-native English-speakers during this period. In contrast, the changing language background of international graduates was found to offset the rise in field-of-study mismatch marginally by 1.5%.

The characteristics effect associated with language background, however, is rather modest compared to other drivers. In particular, the results point to the declining share of international graduates who are an Australian citizen or permanent resident as the key contributing factor for the deterioration in their labour market outcomes. As Table 3 shows, this compositional change explains 39.2% of the rise in economic inactivity between 2000 and 2015, together with an appreciable share of 23.5% for part-time employment and 20.0% for qualification mismatch. It also explains about 30.8% and 14.3% of the growth in field-of-study mismatch and skill mismatch between 2008 and 2015, respectively. These large effects come as no surprise considering the relevance of Australian citizenship and permanent residency in the Australian labour market (Li & Miller, 2013; Blackmore et al., 2014; Gregory, 2014; Hawthorne & To, 2014; Robertson 2014).

The coefficients effect associated with Australian citizenship and permanent residency also played a critical role in the worsening labour market outcomes of international graduates. The results indicate that the coefficients effect contributed 12.1% of the rise in economic inactivity, 25.4% for unemployment, 17.8% for part-time employment, 3.0% for qualification mismatch, and 16.8% for skill mismatch. Nonetheless, an opposite effect was found for field-of-study mismatch. The coefficients effect cushioned the deterioration observed for this labour market indicator by 32.1%. Taken together, these results suggest a decline over time in the protective effect offered by Australian citizenship and permanent residency.^3^ Despite improvements in some aspects, international graduates who hold a temporary visa are increasingly less likely to secure discipline-related work compared to those who are a citizen or permanent resident of Australia. This finding may reflect the growing focus of international graduates with temporary visas on obtaining Australian citizenship or permanent residency, instead of searching for discipline-related job opportunities (Blackmore et al., 2014, 2017).

The results further highlight the characteristics effect and coefficients effect associated with paid work experience during the final year of study as a major force shaping the deterioration in the labour market outcomes of international graduates. As Table 3 shows, the decreasing share of international graduates who took up paid work during the final year of study explains 44.5% and 52.7% of the rise in economic inactivity and unemployment, respectively. This compositional change, however, offset the rise in part-time employment by 6.9%, qualification mismatch by 4.4%, field-of-study mismatch by 4.1%, and skill mismatch by 2.0%. On the other hand, a considerable portion of the rise in part-time employment (12.3%) and qualification mismatch (14.9%) can be traced to the coefficients effect, which in turn buffered 4.5% and 8.3% of the increase in economic inactivity and unemployment, respectively. These results indicate that international graduates who engaged in paid work during the final year of study are increasingly more likely to be economically active and to work after course completion, though mainly in part-time jobs that do not align with their level of education. Collectively, the differing effects across labour market indicators reflect limited access to discipline-related employment opportunities amongst international students and their tendency to take up low-skill jobs, many of which are retained following the completion of their studies (Nyland et al., 2009; Blackmore et al., 2014; Robertson 2014).

Table 3 also presents some interesting results relating to field-of-study choices and unobserved factors. Specifically, 8.9% of the growth in unemployment and 9.1% of the growth in qualification mismatch between 2000 and 2015 is associated with compositional changes in field-of-study choices. The results further show that the characteristics effect mainly resulted from a larger share of international graduates specialising in Management and Commerce, alongside lower interests in Education. Coefficients effects associated with employment location explained a large part of rising field-of-study mismatch (191.7%) and skill mismatch (32.1%) between 2008 and 2015. On the other hand, the model’s constant - which captures the overall effect of contributing factors not included in this study - accounts for 33.3%, 82.0% and 88.6% of the growth in part-time employment, qualification mismatch and field-of-study mismatch, respectively. The largest effect, in percentage term, was found for skill mismatch (111.2%).^4^

## Factors driving the differences in the labour market outcomes of international and domestic graduates

Table 4 presents the summary statistics for the second analysis on the differences in the labour market outcomes of international and domestic graduates. As expected, international graduates were found to underperform in the Australian labour market compared to their domestic counterparts. The differences in their labour market outcomes are especially large in regard to unemployment (22.7% point), part-time employment (25.0% point), and qualification mismatch (23.6% point). The gaps in economic inactivity (10.8% point), field-of-study mismatch (9.7% point) and skill mismatch (4.1% point) are small by comparison. The table also sheds light on differences in their socio-demographic and skill composition. Compared to local graduates, international graduates tend to speak English as an additional language (67.3% point) and lack paid work experience during the final year of study (33.7% point). Furthermore, international graduates tend to hold a higher level of education; more than half had completed a graduate degree, compared to about one-third of domestic graduates. Notwithstanding, international graduates are appreciably over-represented in Management and Commerce (27.2% point), but under-represented in Society and Culture (13.2% point) and Education (12.7% point).

**Fig. 2:**
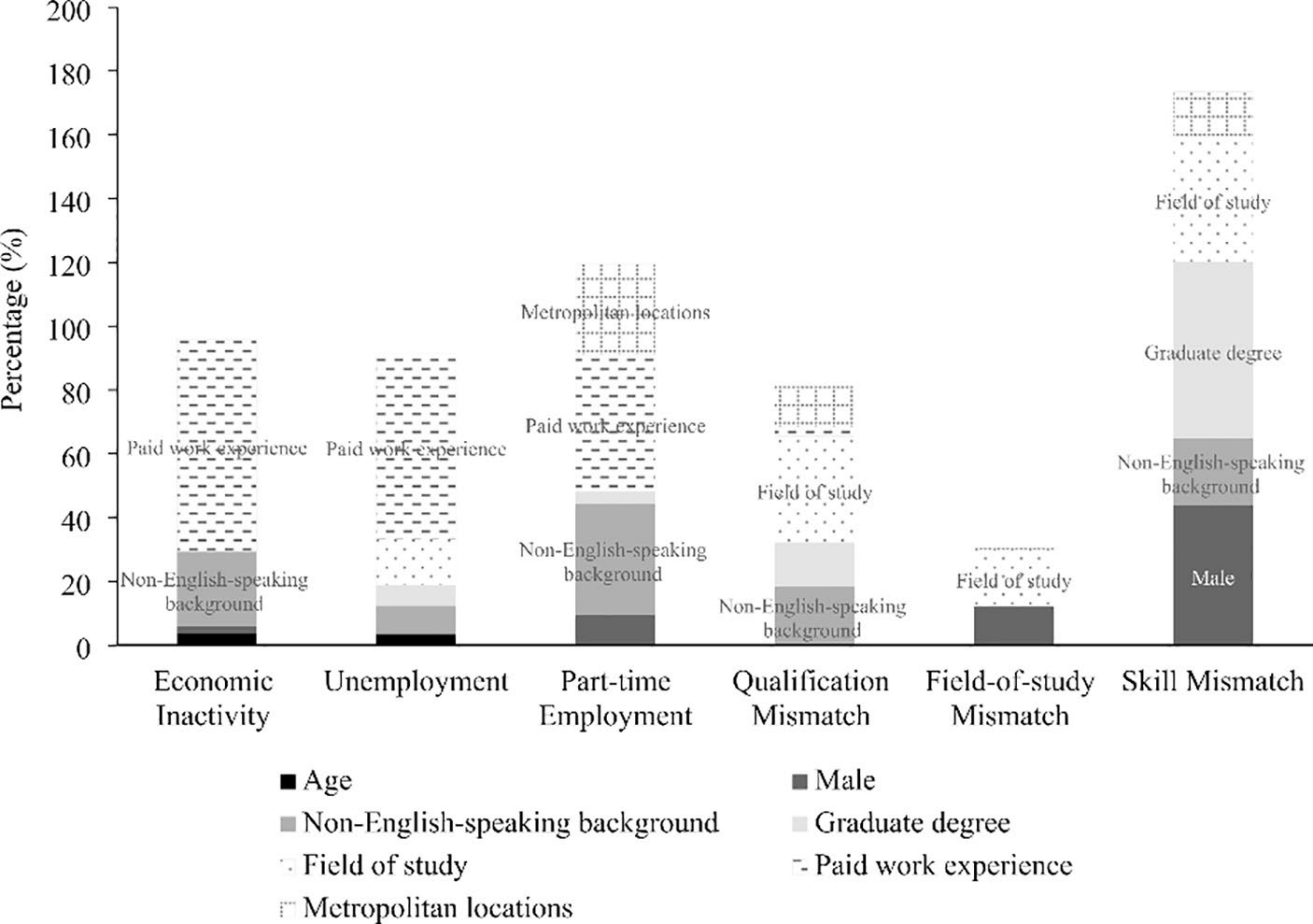
Main factors driving the gaps in the labour market outcomes of international and domestic graduates **Notes** The figure shows the net positive contribution of the main drivers of changes in the respective labour market indicator combining both the characteristics effect and the coefficients effect.

Figure 2 summarises the main factors underpinning the differences in the labour market outcomes of international and local graduates. Of the nine variables included in the models, paid work experience and language background appear to be the main contributing factors explaining a substantial part of the gaps in both economic inactivity and part-time employment. Further, field-of-study choices seem to be another key contributing factor, mainly in relation to qualification mismatch and skill mismatch. Other variables - for example, gender, level of education and employment location - were also important in explaining the differences in the labour market outcomes of international and domestic graduates. Their contributions are nonetheless relatively small, as shown in Fig. 2.

**Table 4:** Summary statistics

Variable	Percentage (%)		Difference (pp)
	**(a)**	**(b)**	**(b) - (a)**
	Domestic	International	International - Domestic
**Dependent variables**			
**Labour market indicators**			
Economic inactivity	4.0	14.8	10.8
Unemployment	10.0	32.7	22.7
Part-time employment	33.3	58.3	25.0
Qualification mismatch	32.4	56.0	23.6
Field-of-study mismatch	23.8	33.5	9.7
Skill mismatch	15.0	19.1	4.1
**Independent variables**			
Age			
*Less than 25*	47.1	34.5	-12.6
*Aged 25–34*	30.1	60.2	30.1
*35 and above*	22.8	5.3	-17.5
Male	36.6	47.3	10.7
Non-English-speaking background	14.2	81.5	67.3
Disability	2.9	1.3	-1.6
Graduate degree	35.3	59.7	24.4
Field of study			
*Sciences and Environmental Studies*	7.1	3.9	-3.2
*Information Technology*	2.8	12.1	9.3
*Engineering and Related Technologies*	6.0	11.2	5.2
*Architecture and Building*	2.7	2.6	-0.1
*Health*	21.5	12.7	-8.8
*Education*	15.8	3.1	-12.7
*Management and Commerce*	19.1	46.3	27.2
*Society and Culture*	18.6	5.4	-13.2
*Creative Arts and Services*	6.5	2.6	-3.9
Unhappy	6.6	6.9	0.3
Paid work experience	81.0	47.3	-33.7
Metropolitan locations	81.8	93.5	11.7

**Notes**: *pp* stands for percentage point. The numbers may not add up due to rounding.

**Source**: Australian Graduate Survey, 2015

Table 4 presents the full decomposition estimation detailing the different factors contributing to differences in the labour market outcomes of international and domestic graduates. As before, the gaps in economic inactivity and unemployment can be broadly traced to compositional differences between international and domestic graduate populations. Meanwhile, the coefficients effects are mainly responsible for the gaps in part-time employment and education-job mismatch. Specifically, the positive characteristics effects associated with non-English-speaking background indicate that the over-representation of non-native English-speaking international graduates contributed to an estimated 22.7% of the difference in economic inactivity, 10.0% for unemployment, 31.8% for part-time employment, 17.9% for qualification mismatch, and 13.6% for skill mismatch. The compositional difference in language background, however, closed the gap in field-of-study mismatch by approximately 14%. The results also highlight compositional difference in the paid work experience of international and domestic graduates as a main contributing factor for the gaps in their labour market outcomes; in fact, the characteristics effect is somewhat large. Specifically, at least half of the gaps in economic inactivity and unemployment could be linked to the smaller share of international graduates who took up paid work during the final year of study. Nevertheless, the positive effect did not extend to other labour market indicators. Instead, the compositional difference was found to narrow the gap in part-time employment by 12.0%, in addition to 8.1% for qualification mismatch, 17.0% for field-of-study mismatch, and 12.1% for skill mismatch. Similarly, these results possibly reflect the tendency of international graduates to take up low-skill jobs while studying and retain these jobs following course completion (Nyland et al., 2009; Blackmore et al., 2014; Robertson 2014).

The gaps in the labour market outcomes of international and local graduates are further attributable to their field-of-study preferences and unobserved characteristics. In particular, compositional differences in field-of-study choices explain an estimated 11.1% and 4.3% of the gaps in unemployment and part-time employment, respectively. This characteristics effect is even greater for education-job mismatch, amounting to 29.7%, 40.6% and 40.2% of the gaps in qualification, field-of-study and skill mismatch, respectively. These positive effects are largely associated with the over-representation of international graduates in Management and Commerce, in conjunction with their under-representation in Education and Health. On the other hand, unobserved characteristics, as captured by the model’s constant, explain an estimated 20.2% of the gap in economic inactivity, along with 9.8% for unemployment, 26.1% for qualification mismatch, and 3.5% for skill mismatch. The greatest effect, in percentage term, was found for field-of-study mismatch (118.8%). This large effect is likely to reflect the influence of citizenship and residency status given its importance in accessing discipline-related work in the Australian labour market (Blackmore et al., 2014) (Table 5).

**Table 5:** Detailed decomposition estimation of the gaps in the labour market outcomes of international and domestic graduates, 2015

Independent variables	Dependent variables											
	**Characteristics effect**						**Coefficients effect**					
	INAC	UNEMP	PTEMP	QMM	FOSMM	SMM	INAC	UNEMP	PTEMP	QMM	FOSMM	SMM
**Aggregate effect**	-65.4	-74.3	25.5	39.5	22.8	55.0	-34.6	-35.7	74.5	60.5	77.2	45.0
**Individual variables**												
Age	-01.3	-00.6	-0.1	-0.1	-1.4	-18.1	-02.3	-02.5	-1.7	-4.9	-2.5	-18.2
*Less than 25*	*0.1*	*-1.6*	*-0.5*	*-1.0*	*-0.5*	*-1.5*	*4.6*	*4.5*	*-6.5*	*-9.5*	*-7.8*	*-28.7*
*Aged 25–34*	*0.9*	*0.1*	*-0.1*	*-0.2*	*-1.0*	*-11.8*	*0.0*	*0.5*	*6.3*	*-0.3*	*5.7*	*-14.9*
*35 and above*	*0.3*	*2.1*	*0.6*	*1.2*	*0.1*	*-4.8*	*-2.2*	*-2.5*	*-1.5*	*4.9*	*-0.4*	*25.4*
Male	0-0.9	-00.5	-0.4	-0.4	6.9	12.4	-03.3	-00.1	9.8	-1.5	5.2	31.5
Non-English-speaking background	-22.7	-10.0	31.8	17.9	-13.8	13.6	-00.7	0-1.3	3.3	0.7	-4.6	7.4
Disability	0-0.3	0-0.1	0.1	0.5	1.1	1.7	0-0.8	0-0.6	-1.0	-1.2	-3.5	-5.3
Graduate degree	0-8.3	-02.3	-4.5	-4.4	-0.9	7.0	0-3.7	-04.0	8.1	17.9	-4.9	48.2
Field of study	0-6.5	-11.1	4.3	29.7	40.6	40.2	-03.7	-03.3	-5.5	3.7	-22.7	-0.8
*Sciences and Environmental Studies*	*0.8*	*-0.4*	*-0.6*	*-1.4*	*-6.6*	*-5.2*	*-1.2*	*-0.8*	*-2.1*	*-1.1*	*-2.4*	*1.4*
*Information Technology*	*-3.0*	*-1.0*	*-2.0*	*0.2*	*14.5*	*-5.2*	*-0.4*	*-0.2*	*0.5*	*0.5*	*1.9*	*-1.0*
*Engineering and Related Technologies*	*-0.9*	*2.1*	*1.0*	*1.0*	*6.7*	*3.9*	*0.2*	*0.8*	*6.0*	*3.6*	*12.4*	*14.1*
*Architecture and Building*	*0.0*	*0.0*	*0.0*	*0.0*	*-0.1*	*0.0*	*0.3*	*-0.1*	*0.2*	*-0.9*	*-0.9*	*-1.6*
*Health*	*1.9*	*2.5*	*1.4*	*5.2*	*22.6*	*20.0*	*1.6*	*4.1*	*-6.7*	*3.2*	*-41.1*	*-5.6*
*Education*	*-2.4*	*6.9*	*-1.3*	*9.8*	*5.9*	*25.0*	*2.1*	*-1.0*	*-3.3*	*3.6*	*6.6*	*-9.5*
*Management and Commerce*	*2.2*	*3.1*	*3.2*	*15.2*	*-25.9*	*14.4*	*1.1*	*1.2*	*11.3*	*6.9*	*16.4*	*16.3*
*Society and Culture*	*-4.8*	*-1.1*	*2.9*	*-0.2*	*18.6*	*-9.4*	*1.0*	*-0.4*	*-7.9*	*-8.9*	*-8.0*	*-6.9*
*Creative Arts and Services*	*-0.4*	*-1.0*	*-0.3*	*0.1*	*4.8*	*-3.3*	*-1.0*	*-0.4*	*-3.5*	*-3.2*	*-7.5*	*-8.2*
Unhappy	-00.0	-00.1	0.0	0.0	-0.1	0.5	0-0.3	0-0.4	0.2	0.3	-1.8	-4.2
Paid work experience	-57.4	-50.0	-12.0	-8.1	-17.0	-12.1	-09.1	-08.4	54.9	10.9	-0.2	-21.3
Metropolitan locations			6.2	4.4	7.2	9.9			22.2	8.5	-6.6	4.1
Constants							-20.2	-09.8	-15.7	26.1	118.8	3.5

**Notes**: The abbreviations (INAC, UNEMP, PTEMP, QMM, FOSMM and SMM) denote, from left to right, economic inactivity, unemployment, part-time employment, qualification mismatch, field-of-study mismatch and skill mismatch. The numbers may not add up due to rounding. The logistic regression coefficients underlying the decomposition analysis are presented in Appendix - Table C.

**Source**: Australian Graduate Survey, 2015

## Discussion and conclusion

Despite their alleged desirability, international graduates appear to experience relatively poor outcomes in the Australian labour market after the completion of their studies, with new evidence pointing to deteriorating outcomes since the initiation of post-study migration and employment pathways in 1999 (Tang et al., 2022). Previous studies have attributed these unfavourable outcomes to a lack of English language skills, permanent residency, work readiness and local discipline-related work experience amongst international graduates (Blackmore et al., 2014, 2017). Nonetheless, the existing literature has not examined the forces driving the deterioration in their labour market outcomes mainly due to the focus on selected subpopulations and specific years. In this respect, we employed Blinder-Oaxaca decompositions to examine the main contributing factors for the worsening labour market outcomes of international graduates since 2000.

The Blinder-Oaxaca decompositions revealed that a considerable part of the deterioration was linked to an influx of non-native English-speaking international graduates. The over-representation of non-native speakers amongst international graduates was also responsible for their disadvantageous labour market position relative to domestic graduates. These results came as no surprise, given poor English language skills amongst recent waves of international graduates (Birrell et al., 2009; Hawthorne 2014). Contrary to expectations, however, the change in the language background and possibly English language skills of international graduates had only a modest effect on their labour market outcomes compared to other contributing factors.

Instead, the decreasing percentage of international graduates who were a citizen or permanent resident of Australia was identified as the key factor contributing to their deteriorating labour market outcomes. While citizenship and permanent residency were found to provide a diminishing protective effect over time, the characteristic remained critical to gain a foothold in the Australian labour market. In particular, the results demonstrated that citizenship and permanent residency were increasingly relevant to securing discipline-related jobs over the period of analysis. In other words, international graduates who remained in Australia with a temporary visa were progressively less likely to obtain work experience in their area of expertise. This finding ironically runs counter to one of the main objectives underpinning the recent policy shift to promote and prioritise temporary retention of international graduates; that is, to provide them an opportunity to accumulate discipline-related work experience in the Australian labour market (Spinks, 2016).

The smaller share of international graduates who took up paid work during the final year of study was also an important contributing factor for their worsening labour market position. The lack of paid work experience amongst recent cohorts of international graduates was shown to account for a large portion of the rise in economic inactivity and unemployment, as was the case for their below-par outcomes compared to local graduates in these domains. However, the dearth in local work experience cushioned the deterioration and widening gaps with respect to part-time employment and education-job mismatch. As pointed out earlier, these contradictions possibly reflect the nature of the jobs typically available to international graduates: low-skill, casual work with limited relevance to their study. The results also underline the tendency of international graduates to remain in these jobs following course completion, an issue that has attracted critical scholarly attention over the years (Jackling, 2007; Birrell and Healy 2008; Li & Miller, 2013; Blackmore et al., 2014, 2017; Hawthorne & To, 2014; Faggian et al., 2016).

Taken together, the present study provides novel quantitative empirical evidence which enhances our understanding of the key forces shaping the labour market outcomes of international graduates in Australia. Drawing on a large sample of international graduates who stayed on since 2000, the study presents additional, robust evidence in relation to the relevance of Australian citizenship and permanent residency, as well as local discipline-related work experience. It demonstrated that language background and English language skills play a less prominent role than suggested by the existing body of qualitative research. The study also offers some insights into the relevance of field-of-study choices, especially in the areas of Management and Commerce, Education and Health.

It is important to acknowledge two limitations in this study. First, the present study was not able to take into account the role of some elements of work readiness, including soft skills and cultural fit - owing to a lack of information on these factors in the AGS. The links between these personal qualities and the deteriorating labour market outcomes of international graduates remain to be elucidated and should be the focus of future research. As pointed out earlier, these largely subjective characteristics might have been used as a subtle exclusion criterion to discriminate against international graduates in the Australian labour market (Blackmore et al., 2014). Second, the present study focused mostly on supply-side factors. In this respect, further research should assess the role of demand-side factors, for example, employers’ attitudes to the employment of international graduates. This additional evidence would yield a better understanding of the labour market standing of international graduates in the Australian economy and society.

Despite these limitations, the present study offers important lessons to policy and practice by challenging existing Australian approaches to international graduate retention. Specifically, our findings reinforced recent findings of the persistent labour market disadvantage experienced by international graduates in Australia. The results further highlighted the inadequacies of some of the existing policies and practices designed to improve the integration of international graduates into the Australian labour market. Amongst other things, the current policies on temporary retention seem to have done little to promote local discipline-related work experience amongst international graduates. These findings call for better interventions to help international graduates integrate into the Australian labour market, including recognising and addressing the structural barriers they face in their transition to work. One of the key structural barriers that warrants immediate policy attention is the differential treatment and stereotypes international graduates have been reported to experience in a labour market that still favours the employment of permanent migrants (Robertson, 2011; Blackmore et al., 2014, 2017; Hawthorne & To, 2014; Tran & Vu, 2016).

The COVID-19 pandemic has drawn further attention to the vulnerability of international graduates - as well as other temporary migrant workers - who were expected to return to their home country when they could no longer financially support themselves against the backdrop of a weak labour market and a lack of government income support (Gibson & Moran, 2020). Specifically, this situation brought to the forefront the uncertainty faced by international graduates who hold a temporary visa. Such uncertainty is one of the major factors that both discourages these graduates from pursuing their careers and employers from hiring them (Blackmore et al., 2014, 2017; Hawthorne & To, 2014; Robertson & Runganaikaloo, 2014). Recently introduced financial incentives and visa extensions may help to attract and retain international graduates in Australia (Brancatisano, 2021; Hawke, 2021). Nonetheless, more action is needed to ensure that these graduates get to encounter a level playing field in the Australian labour market and achieve better integration outcomes.

## Data Availability

The data that support the findings of this study are available from the education department of the Government of Australia. Restrictions apply to the availability of these data, which were used under license for this study.

## Appendix

**Table A:** Description of independent variables for the decomposition analyses

Independent variable	Label	Description
Age	Less than 25	1: Respondent is less than 25 years old; 0: Otherwise
	Aged 25–34	1: Respondent is between 25 and 34 years old; 0: Otherwise
	35 and above	1: Respondent is 35 years old and above; 0: Otherwise
Gender	Male	1: Respondent is male; 0: Respondent is female
Language background	Non-English-speaking background	1: Respondent has a non-English-speaking background; 0: Respondent has an English-speaking background
Disability status	Disability	1: Respondent self-identified to have a disability; 0: Otherwise
Country of permanent residence ^a^	Australia	1: Respondent self-identified as a citizen or permanent resident of Australia; 0: Otherwise
	China	1: Respondent self-identified China as country of permanent residence; 0: Otherwise
	Hong Kong	1: Respondent self-identified Hong Kong as country of permanent residence; 0: Otherwise
	India	1: Respondent self-identified India as country of permanent residence; 0: Otherwise
	Indonesia	1: Respondent self-identified Indonesia as country of permanent residence; 0: Otherwise
	Malaysia	1: Respondent self-identified Malaysia as country of permanent residence; 0: Otherwise
	Singapore	1: Respondent self-identified Singapore as country of permanent residence; 0: Otherwise
	Europe	1: Respondent self-identified a European country as country of permanent residence; 0: Otherwise
	South-East Asia	1: Respondent self-identified a South-East Asian country as country of permanent residence; 0: Otherwise
	North-East Asia	1: Respondent self-identified a North-East Asian country as country of permanent residence; 0: Otherwise
	Southern and Central Asia	1: Respondent self-identified a Southern or Central Asian country as country of permanent residence; 0: Otherwise
	Americas	1: Respondent self-identified an American country as country of permanent residence; 0: Otherwise
	Africa, Middle East and Oceania	1: Respondent self-identified an African, Middle Eastern or Oceanian country as country of permanent residence; 0: Otherwise
Highest level of education ^b^	Graduate degree	1: Respondent self-identified a postgraduate degree, graduate diploma or graduate certificate as the highest level of education 0: Respondent self-identified a bachelor degree, advanced diploma, diploma, certificate or other education as the highest level of education
Field of study ^b^	Sciences and Environmental Studies	1: Respondent self-identified a field in Natural and Physical Sciences or Agriculture, Environmental and Related Studies as major field of study; 0: Otherwise
	Information Technology	1: Respondent self-identified a field in Information Technology as major field of study; 0: Otherwise
	Engineering and Related Technologies	1: Respondent self-identified a field in Engineering and Related Technologies as major field of study; 0: Otherwise
	Architecture and Building	1: Respondent self-identified a field in Architecture and Building as major field of study; 0: Otherwise
	Health	1: Respondent self-identified a field in Health as major field of study; 0: Otherwise
	Education	1: Respondent self-identified a field in Education as major field of study; 0: Otherwise
	Management and Commerce	1: Respondent self-identified a field in Management and Commerce as major field of study; 0: Otherwise
	Society and Culture	1: Respondent self-identified a field in Society and Culture as major field of study; 0: Otherwise
	Creative Arts and Services	1: Respondent self-identified a field in Creative Arts and Services as major field of study; 0: Otherwise
Overall course satisfaction	Unhappy	1: Respondent was dissatisfied or strongly dissatisfied with the quality of the course or higher degree research experience 0: Respondent was strongly satisfied, satisfied or neutral with the quality of the course or higher degree research experience
Paid work experience during the final year of study	Paid work experience	1: Respondent self-identified to have participated in paid work during the final year of study; 0: Otherwise
Employment location ^c^	Metropolitan locations	1: Respondent self-identified to be working in Major Cities of Australia; 0: Respondents self-identified to be working in Inner Regional Australia, Outer Regional Australia, Remote Australia or Very Remote Australia

**Notes**:

^a^ China, Hong Kong, India, Indonesia, Malaysia and Singapore were the top six source countries of international graduates who stayed on as temporary residents between 1998 and 2015, and hence are listed individually. The rest are grouped as per the Major Groups (1-digit level) in the Standard Australian Classification of Countries (ABS 2016b). Americas include Northern, Central and South Americas, as well as the Caribbean. In this study, China refers to the People’s Republic of China, and excludes the Hong Kong Special Administrative Region, the Macau Special Administrative Region, and Taiwan - or formally the Republic of China. Due to small number of observations, North-West Europe and Southern and Eastern Europe are collapsed into a single category, Europe, and Oceania and Antarctica, North Africa and the Middle East and Sub-Saharan Africa into Africa, Middle East and Oceania. This variable only applies to the first analysis on the deteriorating labour market outcomes of international graduates. It is omitted from the second analysis on the gaps between international and domestic graduates, as the latter is not required to identify their country of permanent residence.

^b^ Highest level of education and field of study are categorised in accordance with the Broad Levels (1-digit and 2-digit levels, respectively) in the Australian Standard Classification of Education (ABS 2001). Natural and Physical Sciences and Agriculture, Environmental and Related Studies are collapsed into a single category, Sciences and Environmental Studies, due to small number of observations.

^c^ Employment location is identified using postcodes and grouped by urban hierarchy based on the Remoteness Structure defined in the Australian Statistical Geography Standard (ABS 2018). Two categories (Migratory-Offshore-Shipping and No Usual Address) are omitted from the analyses due to its non-spatial special-purpose nature. Postcodes that extend across two or more urban hierarchies are assigned into the most populous category. Inner Regional Australia, Outer Regional Australia, Remote Australia and Very Remote Australia are collapsed into a single category (non-metropolitan locations) due to small number of observations.

**Table B:** Logistic regression coefficients for the first decomposition analysis

*Logistic regression coefficients - Reference group*						
Independent variables	Dependent variables					
	INAC	UNEMP	PTEMP	QMM	FOSMM	SMM
Age (Reference category: Aged 25–34)						
*Less than 25*	-0.346^***^	0.210^***^	0.360^**^	0.586^***^	-0.114	-0.118
*35 and above*	0.249^***^	0.331^***^	0.373^*^	-0.189	-0.165	-0.005
Male	-0.395^***^	0.068^***^	-0.414^***^	0.009	0.382^***^	0.102
Non-English-speaking background	0.511^***^	0.176^***^	0.698^***^	0.515^***^	0.009	0.119
Disability	0.454^***^	-0.145^***^	0.780^***^	0.787^***^	0.523	0.392
Country of permanent residence (Reference category: Australia)						
*China*	1.946^***^	-0.317^***^	1.425^***^	1.195^**^	0.161	0.223
*Hong Kong*	2.703^***^	1.672^***^	0.372	0.928	0.617^***^	1.148^***^
*India*	2.027^***^	1.561^***^	1.395^**^	-0.686	0.308^**^	0.293^*^
*Indonesia*	1.599^***^	1.717^***^	0.950^*^	0.750	0.405^**^	0.775^***^
*Malaysia*	0.994^***^	1.453^***^	1.291^***^	0.894^**^	0.283^*^	0.427^**^
*Singapore*	1.317^***^	0.796^***^	1.081^***^	-0.475	0.249	0.214
*Europe*	2.183^***^	0.189^***^	0.695	0.158	0.085	0.392^*^
*South-East Asia*	1.868^***^	-0.377^***^	1.742^***^	0.638	0.496^**^	0.509^**^
*North-East Asia*	1.542^***^	0.667^***^	0.897^*^	1.400^***^	0.451^**^	0.728^***^
*Southern and Central Asia*	1.034^***^	1.345^***^	1.758^*^	2.048^**^	0.586^***^	0.423^**^
*Northern, Central and South America*	3.102^***^	0.759^***^	1.077	1.413	0.180	0.209
*Africa, Middle East and Oceania*	1.693^***^	1.104^***^	1.386^**^	-1.058	0.308^*^	0.062
Graduate degree	-0.258^***^	-0.416^***^	0.066	-0.589^***^	0.150^*^	0.155
Field of study (Reference category: Management and Commerce)						
*Sciences and Environmental Studies*	-0.558^***^	-0.011^***^	0.595^**^	0.541^**^	1.027^***^	-0.161
*Information Technology*	-0.810^***^	-0.562^***^	-0.319	-0.783^***^	1.148^***^	0.025
*Engineering and Related Technologies*	-0.617^***^	-0.713^***^	-0.630^*^	-0.403	0.433^***^	-0.281
*Architecture and Building*	-0.336^***^	-0.147^***^	0.198	-0.210	0.399^*^	-1.193^***^
*Health*	-1.363^***^	-1.247^***^	-0.009	-1.011^***^	-0.931^***^	-1.457^***^
*Education*	-0.924^***^	-1.479^***^	0.265	-1.237^***^	0.103	-0.568^*^
*Society and Culture*	-0.454^***^	-0.036^***^	0.486^**^	0.753^***^	0.008	0.335^*^
*Creative Arts and Services*	-0.036^***^	-0.237^***^	0.708^***^	-0.250	0.123	0.228
Unhappy	0.224^***^	0.037^***^	0.184	-0.010	0.008	0.537^***^
Paid work experience	-1.517^***^	-1.942^***^	0.322	-0.031	0.303^***^	0.174^*^
Metropolitan locations			0.218	1.333^***^	-0.230	0.227
*N*	3,358^***^	3,238^***^	2,213	2,198	4,459	4,006
pseudo *R*^2^	0.231^***^	0.185^***^	0.069	0.122	0.078	0.047

**Notes**: The abbreviations (INAC, UNEMP, PTEMP, QMM, FOSMM and SMM) denote, from left to right, economic inactivity, unemployment, part-time employment, qualification mismatch, field-of-study mismatch and skill mismatch.

**Source**: Australian Graduate Survey, 2000 and 2008

**Significance levels**: ^*^*p* < 0.1, ^**^*p* < 0.05, ^***^*p* < 0.01

**Table:** *Logistic regression coefficients - Comparison group*

Independent variables	Dependent variables					
	INAC	UNEMP	PTEMP	QMM	FOSMM	SMM
Age (Reference category: Aged 25–34)						
*Less than 25*	-0.122^***^	0.116^***^	0.003	0.081	-0.004	0.141
*35 and above*	0.044^***^	-0.133^***^	0.025	-0.109	-0.034	0.166
Male	-0.044^***^	0.100^***^	-0.111	-0.120	0.242^***^	0.338^***^
Non-English-speaking background	0.288^***^	0.114^***^	0.314^***^	0.222^**^	-0.103	0.147
Disability	0.149^***^	0.061^***^	-0.095	-0.350	-0.372	-0.423
Country of permanent residence (Reference category: Australia)						
*China*	1.158^***^	0.240^***^	0.747^***^	0.270^**^	0.298^**^	-0.364^**^
*Hong Kong*	1.105^***^	0.132^***^	0.911^***^	0.626^***^	0.985^***^	0.510^**^
*India*	0.238^***^	-0.257^***^	1.007^***^	0.981^***^	0.857^***^	0.547^***^
*Indonesia*	0.811^***^	0.307^***^	0.839^***^	0.386^*^	0.781^***^	0.667^***^
*Malaysia*	1.016^***^	0.078^***^	0.377^**^	0.394^**^	0.487^**^	0.420^*^
*Singapore*	1.037^***^	-0.083^***^	0.308	0.108	0.646^**^	0.114
*Europe*	0.878^***^	-0.325^***^	0.048	0.296	0.500^**^	0.452^*^
*South-East Asia*	0.815^***^	0.091^***^	0.939^***^	0.793^***^	0.756^***^	0.199
*North-East Asia*	0.915^***^	0.196^***^	0.524^***^	0.311	0.817^***^	0.178
*Southern and Central Asia*	0.524^***^	-0.149^***^	0.986^***^	0.911^***^	0.678^***^	0.444^**^
*Northern, Central and South America*	0.551^***^	-0.285^***^	-0.117	0.207	0.535^***^	0.450^**^
*Africa, Middle East and Oceania*	1.078^***^	0.129^***^	0.886^***^	0.797^***^	0.720^***^	0.073
Graduate degree	-0.309^***^	0.159^***^	-0.192^**^	-0.200^**^	0.016	0.120
Field of study (Reference category: Management and Commerce)						
*Sciences and Environmental Studies*	-0.360^***^	0.075^***^	0.135	-0.069	1.583^***^	0.464^**^
*Information Technology*	-0.324^***^	-0.219^***^	-0.467^***^	-0.755^***^	0.988^***^	-0.494^***^
*Engineering and Related Technologies*	-0.255^***^	0.406^***^	0.195	-0.343^***^	1.343^***^	0.190
*Architecture and Building*	0.146^***^	-0.265^***^	-0.277	-0.555^***^	0.985^***^	-0.442
*Health*	-0.262^***^	-0.492^***^	-0.351^***^	-1.387^***^	-0.932^***^	-1.188^***^
*Education*	0.116^***^	-0.871^***^	0.181	-1.301^***^	0.309^*^	-0.951^***^
*Society and Culture*	0.373^***^	-0.004^***^	-0.255^*^	-0.544^***^	-0.184	0.014
*Creative Arts and Services*	0.052^***^	0.251^***^	0.019	-0.617^***^	-0.227	0.114
Unhappy	-0.081^***^	0.204^***^	0.044	0.076	-0.161	0.633^***^
Paid work experience	-1.869^***^	-2.218^***^	0.679^***^	0.413^***^	0.367^***^	0.186^*^
Metropolitan locations			0.473^***^	0.334^**^	0.221	0.290
*N*	8,319^***^	7,089^***^	4,193	3,913	3,932	3,729
pseudo *R*^2^	0.128^***^	0.205^***^	0.058	0.073	0.107	0.061

**Notes**: The abbreviations (INAC, UNEMP, PTEMP, QMM, FOSMM and SMM) denote, from left to right, economic inactivity, unemployment, part-time employment, qualification mismatch, field-of-study mismatch and skill mismatch.

**Source**: Australian Graduate Survey, 2015

**Significance levels**: ^*^*p* < 0.1, ^**^*p* < 0.05, ^***^*p* < 0.01

**Table C:** Logistic regression coefficients for the second decomposition analysis

*Logistic regression coefficients - Reference group*						
Independent variables	Dependent variables					
	INAC	UNEMP	PTEMP	QMM	FOSMM	SMM
Age (Reference category: Aged 25–34)						
*Less than 25*	-0.298^***^	-0.080^***^	0.423^***^	0.322^***^	0.183^***^	0.221^***^
*35 and above*	0.188^***^	0.235^***^	0.265^***^	-0.336^***^	0.099^***^	-0.252^***^
Male	-0.329^***^	0.057^***^	-0.344^***^	0.010	0.233^***^	0.148^***^
Non-English-speaking background	0.245^***^	0.498^***^	0.198^***^	0.198^***^	0.070^**^	-0.115^***^
Disability	0.845^***^	0.811^***^	0.403^***^	0.248^***^	0.232^***^	0.222^***^
Graduate degree	-0.110^***^	-0.230^***^	-0.449^***^	-0.789^***^	0.041	-0.292^***^
Field of study (Reference category: Management and Commerce)						
*Sciences and Environmental Studies*	0.188^***^	0.562^***^	1.083^***^	0.634^***^	2.181^***^	0.779^***^
*Information Technology*	0.083^***^	0.186^***^	0.087	-0.365^***^	1.120^***^	0.000
*Engineering and Related Technologies*	-0.205^***^	0.191^***^	-0.306^***^	-0.652^***^	0.727^***^	-0.313^***^
*Architecture and Building*	0.022^***^	0.040^***^	0.149^**^	0.216^***^	1.427^***^	0.007
*Health*	-0.381^***^	-0.941^***^	0.616^***^	-0.992^***^	0.309^***^	-0.731^***^
*Education*	-0.073^***^	-0.501^***^	0.822^***^	-1.223^***^	0.434^***^	-0.479^***^
*Society and Culture*	0.320^***^	0.215^***^	0.726^***^	0.452^***^	0.380^***^	0.434^***^
*Creative Arts and Services*	0.544^***^	0.604^***^	1.234^***^	0.486^***^	0.758^***^	0.850^***^
Unhappy	0.033^***^	0.373^***^	-0.056	0.000	-0.026	0.860^***^
Paid work experience	-2.204^***^	-2.549^***^	-0.031	0.269^***^	0.378^***^	0.286^***^
Metropolitan locations			0.246^***^	0.237^***^	0.295^***^	0.274^***^
*N*	62,342^***^	59,820^***^	49,449	47,062	47,148	45,962
pseudo *R*^2^	0.155^***^	0.231^***^	0.055	0.129	0.064	0.070

**Notes**: The abbreviations (INAC, UNEMP, PTEMP, QMM, FOSMM and SMM) denote, from left to right, economic inactivity, unemployment, part-time employment, qualification mismatch, field-of-study mismatch and skill mismatch.

**Source**: Australian Graduate Survey, 2015

**Significance levels**: ^*^*p* < 0.1, ^**^*p* < 0.05, ^***^*p* < 0.01

**Table:** *Logistic regression coefficients - Comparison group*

Independent variables	Dependent variables					
	INAC	UNEMP	PTEMP	QMM	FOSMM	SMM
Age (Reference category: Aged 25–34)						
*Less than 25*	-0.043^***^	0.160^***^	0.046	0.086	0.031	0.180^*^
*35 and above*	-0.055^***^	-0.171^***^	-0.033	-0.063	0.011	0.238
Male	-0.095^***^	0.064^***^	-0.047	-0.040	0.296^***^	0.419^***^
Non-English-speaking background	0.379^***^	0.201^***^	0.490^***^	0.269^***^	-0.089	0.072
Disability	0.186^***^	0.097^***^	-0.076	-0.382	-0.404	-0.472
Graduate degree	-0.382^***^	0.121^***^	-0.210^***^	-0.199^**^	-0.017	0.109
Field of study (Reference category: Management and Commerce)						
*Sciences and Environmental Studies*	-0.385^***^	0.010^***^	0.102	-0.008	1.643^***^	0.585^***^
*Information Technology*	-0.453^***^	-0.299^***^	-0.345^***^	-0.581^***^	1.070^***^	-0.381^***^
*Engineering and Related Technologies*	-0.286^***^	0.376^***^	0.183	-0.317^**^	1.308^***^	0.192
*Architecture and Building*	0.193^***^	-0.259^***^	-0.392^**^	-0.633^***^	0.906^***^	-0.431
*Health*	-0.332^***^	-0.551^***^	-0.332^***^	-1.268^***^	-0.822^***^	-1.068^***^
*Education*	0.119^***^	-0.895^***^	-0.027	-1.398^***^	0.234	-0.922^***^
*Society and Culture*	0.315^***^	-0.049^***^	-0.371^***^	-0.586^***^	-0.181	0.054
*Creative Arts and Services*	0.011^***^	0.213^***^	-0.035	-0.631^***^	-0.177	0.150
Unhappy	-0.103^***^	0.178^***^	-0.028	0.057	-0.151	0.653^***^
Paid work experience	-1.914^***^	-2.233^***^	0.645^***^	0.418^***^	0.377^***^	0.212^**^
Metropolitan locations			0.541^***^	0.363^***^	0.260	0.289
*N*	8,319^***^	7,089^***^	4,193	3,913	3,932	3,729
pseudo *R*^2^	0.111^***^	0.200^***^	0.035	0.057	0.096	0.044

**Notes**: The abbreviations (INAC, UNEMP, PTEMP, QMM, FOSMM and SMM) denote, from left to right, economic inactivity, unemployment, part-time employment, qualification mismatch, field-of-study mismatch and skill mismatch.

**Source**: Australian Graduate Survey, 2015

**Significance levels**: ^*^*p* < 0.1, ^**^*p* < 0.05, ^***^*p* < 0.01

## Footnotes

1 The AGS was replaced by the Graduate Outcomes Survey (GOS) in 2016. While the GOS continues to collect data on the labour market outcomes and job search behaviour of recently qualified tertiary graduates in Australia, it has incorporated numerous important changes to question wording, coding scheme and survey reference period. For example, the AGS captured labour market outcomes and job search behaviour on 31 October for the first main round and 30 April for the second round; in contrast, the GOS refers to the week before survey for labour market outcomes and the past four weeks for job search behaviour. Owing to these changes, the AGS and the GOS are not directly comparable, limiting our scope of analysis to the 18 years between 1998 and 2015. Given the period of analysis, we will refer to the survey as the AGS in this article.

2 The methodology proposed by Yun (2004, 2008) addresses two issues commonly linked to non-linear detailed decomposition analysis: the identification problem - which concerns the choice of reference groups for sets of dummy variables - and path dependence - which concerns the order in which predictors are introduced into the model (Powers et al., 2011).

3 Nevertheless, international graduates who had attained Australian citizenship or permanent residency were still at an advantage in the Australian labour market, compared to those with a temporary visa (see Appendix B).

4 It is important to bear in mind that the increases in field-of-study mismatch and skill mismatch were relatively small − 5.3 and 3.0% points, respectively - which made the aforementioned percentages seem overly large.

## Code availability

Not available due to restrictions on the availability of the data.
